# Cognitive and Neuropsychiatric features of a Frontotemporal Dementia Cohort in Peruvian Population

**DOI:** 10.1002/alz70857_107720

**Published:** 2025-12-25

**Authors:** Sheila Castro‐Suarez, Jonathan Adrian Zegarra‐Valdivia, Erik Alberto Guevara‐Silva, Cesar Caparo‐Zamalloa, Henry Palomino‐Lescano, José Cuenca, Kelvin Alvarez‐Toledo, Maryenela Illanes‐Manrique, Nilton Custodio, Serggio Lanata, Maria Meza‐Vega, Mario Cornejo‐Olivas

**Affiliations:** ^1^ Atlantic Fellow for Equity in Brain Health at Global Brain Health Institute (GBHI), San Francisco, CA, Peru; ^2^ CBI en Demencias y Enfermedades Desmielinizantes del Sistema Nervioso, Instituto Nacional de Ciencias Neurológicas, Lima, Peru; ^3^ Global Brain Health Institute (GBHI), University of California San Francisco, San Francisco, CA, USA; ^4^ Universidad Señor de Sipán, Chiclayo, Peru; ^5^ CBI en Demencia y Enfermedades Desmielinizantes del Sistema Nervioso, Lima, Lima, Peru; ^6^ Universidad Cientifica de Sur, Lima, Lima, Peru; ^7^ CBI en Demencias y Enfermedades Desmielinizantes del Sistema Nervioso, Instituto Nacional de Ciencias Neurologicas, Lima, Peru; ^8^ Atlantic Fellow for Equity in Brain Health at Global Brain Health Institute (GBHI), San Francisco, CA, USA; ^9^ Neurogenetics Working Group, Universidad Cientifica del Sur, Lima, Peru; ^10^ Unit Cognitive Impairment and Dementia Prevention, Peruvian Institute of Neurosciences, Lima, Peru, Lima, Lima, Peru; ^11^ Unidad de Investigación, Instituto Peruano de Neurociencias, Lima, Lima, Peru; ^12^ Global Brain Health Institute, University of California, San Francisco, San Francisco, CA, USA; ^13^ Memory and Aging Center, UCSF Weill Institute for Neurosciences, University of California, San Francisco, San Francisco, CA, USA; ^14^ School of Medicine, Universidad Nacional Mayor de San Marcos, Lima, Peru; ^15^ Neurogenetics Research Center, Instituto Nacional de Ciencias Neurológicas, Lima, Peru

## Abstract

**Background:**

The prevalence of frontotemporal dementia (FTD) is 1.9% over 65 years. There is a considerable delay in the diagnosis of FTD and a high rate of misdiagnosis in Latin America. We describe cognitive and neuropsychiatric features in a Peruvian cohort.

**Method:**

Participants have been recruited using Lima FTD network and then they were assessed by neurologists, neuropsychologists, and psychiatrists. The participants completed comprehensive neuropsychological battery, neuropsychiatric and functional assessments. IRB approval was obtained from CIEI‐INCN.

**Result:**

We studied 18 FTD cases and 39 matched controls. The FTD group had 72.22% males, while the control group had 53.85%. The mean age at onset in the FTD group was 55.94 ± 9.24 years, and at evaluation it was 58.61 ± 8.83 years while in control group the mean age was 61.54 ± 5.92 years. FTD patients had 13.11 ± 3.61 years of education, significantly higher than 7.33 ± 1.15 years in controls (*p* = 0.000). Diagnoses were: 16 (88.9%) BvFTD and 2 (11.1%) semantic PPA. Most common symptoms included dysexecutive dysfunction (100%), disinhibition, apathy, and perseverative behavior (88.9% each). ACE scores were much lower in FTD (34.88 ± 21.39) than controls (83.26 ± 17.21, *p* =  0.000), as were INECO frontal screening (5.3 ± 4.08 vs. 17.18 ± 4.6, *p* =  0.000) and Digit Forward scores (3.5 ± 2.22 vs. 7.05 ± 2.01, *p* =  0.000). According to the NPI, the most common symptoms were disinhibition (88.2%), apathy (82.4%), and aberrant motor behavior (76.5%). Dementia severity was rated using CDR: 16.7% had CDR=3, 66.7% had CDR=2, and 16.7% had CDR=1. Depressive symptoms averaged 3.8 ± 2.7 on the GDS. Correlations were not significant between years of education and cognitive performance or between GDS and CDR scores.

**Conclusion:**

The clinical features of the FTD cohort in the Peruvian population are consistent with previous reports. Among the Peruvian FTD cohort, the main neuropsychiatric symptoms are disinhibition, apathy, and perseverative behavior

